# Comparative safety of denosumab and romosozumab in osteoporosis: an analysis based on the FDA adverse event reporting system database

**DOI:** 10.3389/fmed.2026.1766601

**Published:** 2026-02-05

**Authors:** Jian Sun, Ciren Lunzhu, Yao Wu, Jun Li

**Affiliations:** 1 Department of Orthopedics, The Second Affiliated Hospital of Anhui Medical University, Hefei, Anhui Province, China; 2 Institute of Orthopedics, Research Center for Translational Medicine, The Second Affiliated Hospital of Anhui Medical University, Hefei, Anhui Province, China; 3 Department of Orthopedics, Shannan City People’s Hospital, Shannan, Xizang Autonomous Region, China; 4 Department of Plastic and Cosmetic Burns, Anhui No.2 Provincial People’s Hospital, Hefei,Anhui Province, China

**Keywords:** denosumab, FAERS, immunology, osteoporosis, pharmacovigilance, romosozumab

## Abstract

**Objective:**

Denosumab and romosozumab are two biological agents with potential immunomodulatory effects commonly used to treat osteoporosis. This study aimed to compare the safety of these two drugs using the FDA Adverse Event Reporting System (FAERS) with a focus on immune-related adverse reactions.

**Methods:**

Data for the second quarter of 2019 (approval date for romosozumab) and the second quarter of 2025 were extracted from the FAERS database. After standardized data cleaning, disproportionality analysis and the Bayesian approach were applied to detect safety signals for the two biological agents simultaneously.

**Results:**

After data extraction and cleaning, 38,784 and 10,007 reports were obtained for denosumab and romosozumab, respectively. Denosumab was associated with long-term cumulative risk and showed strong signals in musculoskeletal and metabolic disorders. In contrast, romosozumab demonstrated an acute and severe cardiovascular risk with strong signals for coronary artery disease and heart failure. Regarding immune-related events, denosumab was associated with osteoimmune complications such as noninfectious gingivitis, whereas the immune-related risk of romosozumab was limited to minor injection-site reactions.

**Conclusion:**

Research based on FAERS revealed distinct differences in the safety profiles of denosumab and romosozumab. Clinical decision makers should deepen their understanding of these features to advance personalized therapy and improve medication safety.

## 1 Introduction

Osteoporosis is a systemic skeletal disease characterized by low bone mass and microarchitectural deterioration of the bone tissue, leading to increased bone fragility and a consequent increase in fracture risk (1). With the accelerated aging of the global population, osteoporosis and related fractures have become major public health concerns (2). In recent years, biological agents have become key options for the treatment of osteoporosis, with denosumab and romosozumab being the two main agents (3).

Denosumab, a fully human monoclonal antibody, reduces osteoclast-mediated bone resorption by inhibiting the receptor activator of nuclear factor kappa-B ligand (RANKL) (4). Its antifracture efficacy has been established in several large-scale randomized controlled trials (4, 5). Notably, RANKL is expressed on various immune cells such as T and dendritic cells (6). Consequently, denosumab possesses defined immunomodulatory potential, and its association with immune-related adverse events, including serious infections and cellulitis, has been investigated in epidemiological studies and meta-analyses (7, 8).

In contrast, romosozumab increases bone mineral density by promoting bone formation and suppressing bone resorption by antagonizing the inhibition of the Wnt signaling pathway by sclerostin (9). Although its primary target appears to be the skeletal system, studies have reported that sclerostin can influence macrophage and cytokine activity (10, 11), implying that romosozumab may possess an underinvestigated immune-related safety profile.

Denosumab inhibits bone resorption via RANKL blockade, whereas romosozumab promotes bone formation via sclerostin inhibition. Both drugs have been reported to possess immunomodulatory potential. In addition, their safety profiles, particularly regarding immune-related and cardiovascular adverse events, remain areas of ongoing clinical concern.

Studies comparing the safety of these two biological agents are limited. This study aimed to leverage the United States Food and Drug Administration (FDA) Adverse Event Reporting System (FAERS) database (12) to systematically describe and compare the safety signal patterns of denosumab and those of romosozumab, with a focus on immune-related adverse reactions, thereby providing evidence for risk-benefit assessment in clinical decision-making.

## 2 Materials and methods

### 2.1 Data extraction and processing

The data used in this study were sourced from public releases of the FAERS database. We extracted data from the time of romosozumab’s initial approval in the United States (Q2 2019) to the second quarter of 2025. Key data, including patient demographic characteristics, drug information, adverse events, reporting sources, and outcomes, were extracted from quarterly FAERS data files. A rigorous and standardized data cleaning process was performed to prepare the data for analysis (13). All quarterly ASCII files were imported into R software and vertically concatenated to create unified tables for the entire study period. Duplicate reports were identified and removed from the DEMO table using the FDA-recommended algorithm based on the primary and case-id fields. Records with the latest FDA receipt dates (FDA_DT) were retained. This deduplication process was first applied to the DEMO table, and the resulting unique primary list was then used to filter the corresponding records in the DRUG, REAC, OUTC, RPSR, THER, and INDI tables, ensuring a non-duplicated dataset across all domains. Potential generic and brand names for denosumab (e.g., “Prolia,” “Xgeva”) and romosozumab (e.g., “Evenity”) were compiled from authoritative sources including Drugs@FDA. These terms were used to search the drug name and prod_ai fields in the DRUG table. Reports where either denosumab or romosozumab was recorded with a role_cod of “PS” (Primary Suspect) were selected for inclusion in the respective drug cohorts. Reports with obvious spelling errors in the drug name were reviewed and either manually corrected or excluded if unambiguous identification was not possible. Key demographic and outcome variables were recorded for consistency. For example, age was grouped into predefined categories (e.g., 18–64, 65–85, and >85 years). Missing values for age, sex, and outcome were explicitly defined and labeled as “Missing/Not Reported” in the final analytic dataset to ensure transparency in the descriptive summaries.

### 2.2 Data analysis and signal detection

Prior to formal analysis, we performed exploratory data analysis to understand the structure, completeness, and potential issues within the raw FAERS data. First, we plotted the quarterly volumes of all reports from Q2 2019 to Q2 2025 to identify any obvious temporal trends or data entry anomalies. Second, we calculated the proportion of missing values for critical variables, including patient age, sex, reporter type, and outcome, stratified by the drug of interest (denosumab or romosozumab), where applicable. Third, we examined the distribution of reports by geographic region and reporting source to identify major imbalances that could influence signal detection. For the disproportionality analysis, all reports in which denosumab or romosozumab was listed as the “PS” were included irrespective of the availability of demographic information. Adverse event terms were coded using the Medical Dictionary for Regulatory Activities version 28.0. For signal detection, an initial screening was performed using the Reporting Odds Ratio (ROR) and Proportional Reporting Ratio (PRR), with signals defined by a lower limit of the 95% confidence interval >1 coupled with a case count of ≥3 for ROR, and a PRR ≥ 2, *χ*^2^ statistic ≥ 4, and case count of ≥3 for PRR. The signals were confirmed using a Bayesian-based Multi-Item Gamma Poisson Shrinker and Bayesian Confidence Propagation Neural Network (BCPNN) algorithms. A confirmed significant signal was defined as one that concurrently met the criteria of an Empirical Bayesian Geometric Mean (EBGM)_05_ > 2 for a Multi-Item Gamma Poisson Shrinker and IC_025_ > 0 for the BCPNN. All statistical analyses and data cleaning were conducted using the R software (version 4.5.1). The Bayesian methods (EBGM and IC) were implemented using the OpenEBGM package. Frequentist methods (ROR and PRR) were calculated using custom R functions based on the standard formulas for the odds ratio and proportion ratio, together with their 95% confidence intervals (for ROR) and χ^2^ statistics (for PRR). The calculation formulas for these indices are well-established; we refer to seminal publications for ROR (14), PRR (15), and Bayesian methods (16).

## 3 Results

### 3.1 Study cohort

**Figure 1:**
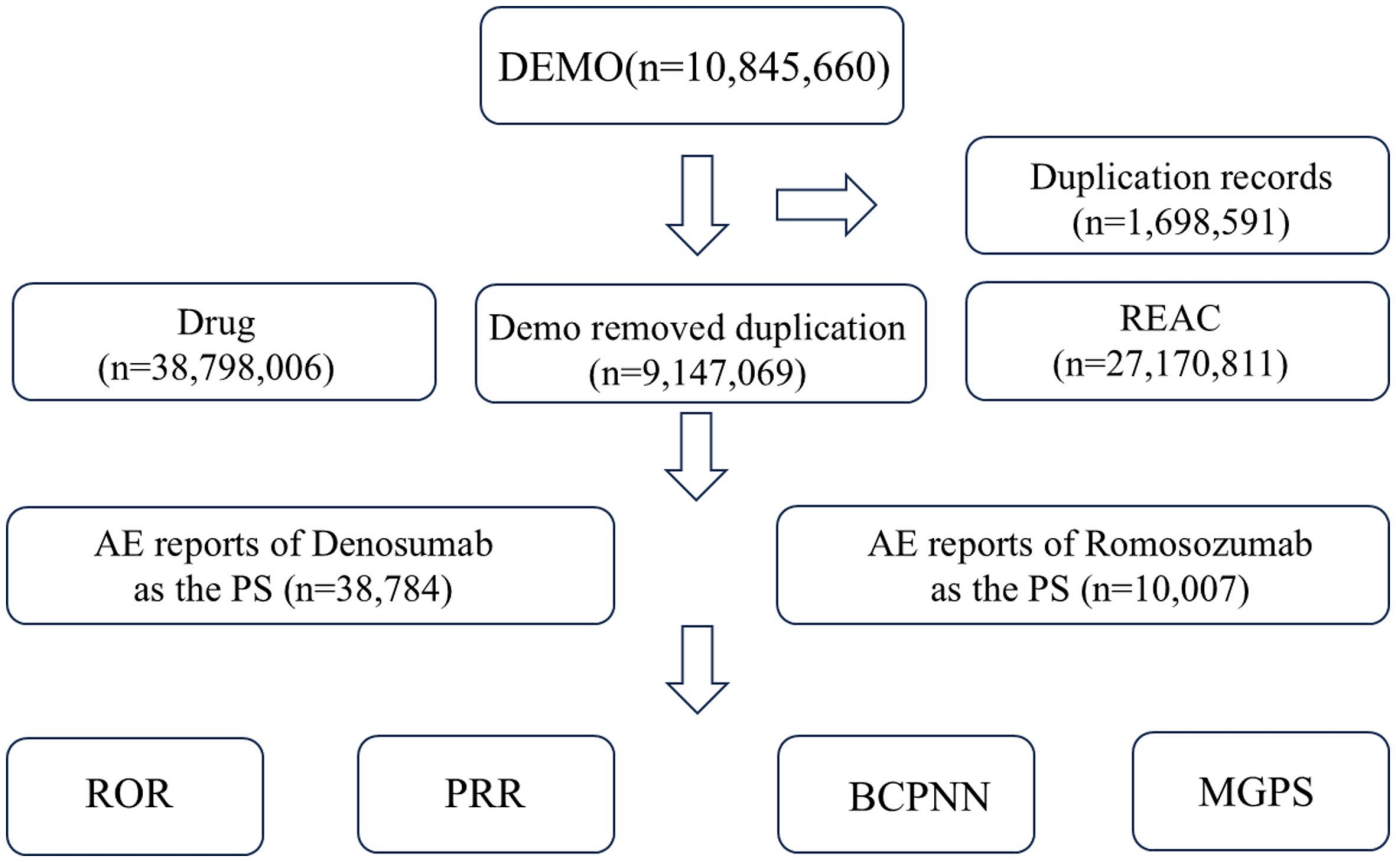
Flowchart of report selection from the FDA Adverse Event Reporting System (FAERS) database for denosumab and romosozumab. Signal detection criteria: Reporting odds ratio (ROR) with lower 95% confidence interval (CI) > 1 and *N* ≥ 3, or proportional reporting ratio (PRR) ≥ 2, *χ*^2^ ≥ 4, and *N* ≥ 3. The multi-item gamma Poisson shrinker (MGPS) required an empirical Bayesian geometric mean 05% lower bound (EBGM05) > 2, and the Bayesian confidence propagation neural network (BCPNN) required an information component 025% lower bound (IC025) > 0.

In total, 10,845,660 initial reports were identified after extracting data from the FAERS database for the period from Q2 2019 to Q2 2025. After data cleaning, 9,147,069 reports were retained for subsequent analysis. From the cleaned dataset, 38,784 reports on denosumab and 10,007 reports on romosozumab were identified as “PS.” The ratio in the report volume was nearly 4:1, possibly owing to the longer market presence of denosumab and, consequently, greater cumulative patient exposure. A flow diagram of the report selection process is shown in Figure 1.

**Figure 2:**
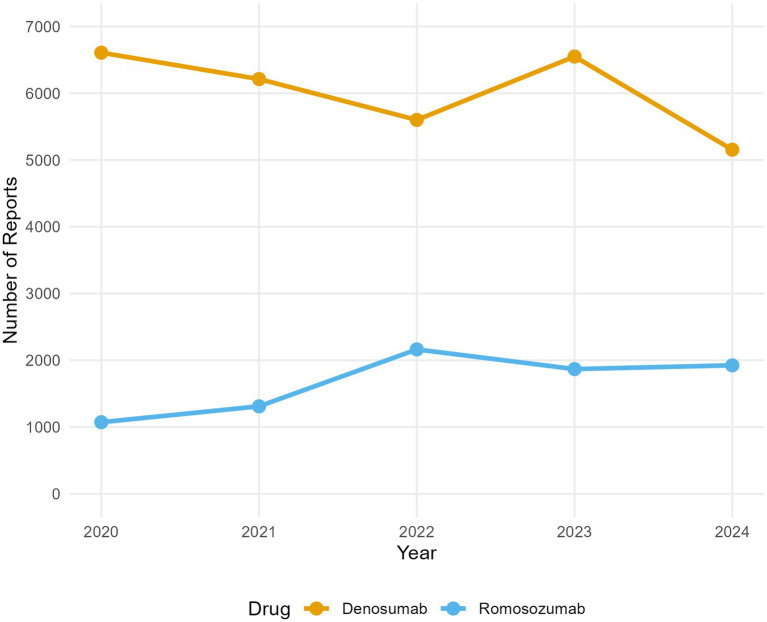
Annual trends in adverse event reports for denosumab and romosozumab in the FDA Adverse Event Reporting System (FAERS) database. The analysis includes data from full reporting years (2020–2024).

The number of reports on each drug from 2019 to 2025 is shown in Table 1. Because romosozumab was launched in Q2 2019 and the FAERS data were updated only through Q2 2025 at the time of study initiation, the datasets for 2019 and 2025 were incomplete. Therefore, data from 2020 to 2024 were used to plot the annual reporting trends (Figure 2). During this period, reports on denosumab treatment consistently exceeded those on romosozumab treatment. Denosumab reports demonstrated a stable pattern, fluctuating between approximately 5,150 and 6,600 reports per year. In comparison, romosozumab reports increased by 102% from 2020 (*n* = 1,073), peaked in 2022 (*n* = 2,163), and subsequently plateaued with only slight variations in 2023 and 2024. This pattern is characteristic of newly marketed drugs and reflects the initial market uptake, followed by stabilization.

**Table 1:** Annual distribution of adverse event reports for denosumab and romosozumab from the FDA adverse event reporting system (FAERS) database (Q2 2019–Q2 2025).

Year	Denosumab		Romosozumab	
	Reports, *n*	Proportion, %	Reports, *n*	Proportion, %
2019	6,385	16.5	607	6.1
2020	6,608	17.0	1,073	10.7
2021	6,213	16.0	1,311	13.1
2022	5,601	14.4	2,163	21.6
2023	6,550	16.9	1,869	18.7
2024	5,155	13.3	1,925	19.2
2025	2,272	5.9	1,059	10.6
Total	38,784	100.0	10,007	100.0

### 3.2 Geographic and demographic distribution

Geographically, denosumab (68.4%) and romosozumab (53.0%) were most frequently reported in the United States, followed by Japan. Notably, Japan accounted for 38.6% of romosozumab reports but only 6.5% of denosumab reports (Supplementary Figure S1). Table 2 lists all the countries that contributed to at least 1% of the reports.

**Table 2:** Geographic distribution of adverse event reports for denosumab and romosozumab from the FDA adverse event reporting system (FAERS) database.

Reporter country	Denosumab, *n* (%)	Romosozumab, *n* (%)
United States	26,519 (68.4)	5,301 (53.0)
Japan	2,509 (6.5)	3,862 (38.6)
Australia	928 (2.4)	26 (0.3)
Canada	906 (2.3)	61 (0.6)
Spain	773 (2.0)	33 (0.3)
Germany	772 (2.0)	95 (0.9)
United Kingdom	558 (1.4)	119 (1.2)
France	518 (1.3)	1 (0.0)
China	489 (1.3)	3 (0.0)
Netherlands	320 (0.8)	116 (1.2)
All other countries	3,492 (9.0)	390 (3.9)
Total	38,784 (100.0)	10,007 (100.0)

Female patients constituted the majority of cases for both denosumab (67.4%) and romosozumab (70.6%), consistent with the higher prevalence of postmenopausal osteoporosis among women. Age distributions were similar between the two cohorts, with the highest proportion reported in individuals aged 65–85 years (denosumab, 30.3%; romosozumab, 28.0%), representing the population at the greatest risk for osteoporotic fractures. A substantial proportion of the reports lacked age data (denosumab, 52.8%; romosozumab, 58.8%), which is a common limitation of spontaneous reporting systems. The reported distributions according to sex and age are shown in Table 3, with a bar chart showing the differences between the two drugs (Supplementary Figure S2).

**Table 3:** Demographic characteristics of patients with adverse event reports for denosumab and romosozumab.

Characteristic	Category	Denosumab, n (%)	Romosozumab, n (%)
Sex	Female	26,128 (67.4)	7,066 (70.6)
	Male	3,794 (9.8)	548 (5.5)
	Missing/not reported	8,862 (22.8)	2,393 (23.9)
Age group	18–64 years	4,342 (11.2)	638 (6.4)
	65–85 years	11,761 (30.3)	2,802 (28.0)
	>85 years	2,024 (5.2)	681 (6.8)
	12–17 years	107 (0.3)	0 (0.0)
	2–11 years	71 (0.2)	3 (0.0)
	<2 years	6 (0.0)	0 (0.0)
	Missing/not reported	20,473 (52.8)	5,883 (58.8)

The category “Missing / Not Reported” includes reports where the age or sex field was blank, contained non-informative entries (e.g., “unknown”), or could not be reliably derived from the original data.

### 3.3 Reporting sources and patient outcomes

Reporting sources differed significantly between the two drugs, with consumers being the most common reporters of denosumab (36.4%), and physicians accounting for most reports related to romosozumab (42.4%). Hospitalization was the most frequently reported serious outcome for both denosumab (9.4%) and romosozumab (19.1%) (Table 4), although the rate for romosozumab was approximately twice as high. Denosumab has a longer market history and patients receiving long-term treatment may be more proactive in spontaneous reporting. In contrast, romosozumab is associated with a high proportion of inpatient outcomes and increased involvement of healthcare professionals in reporting adverse events.

**Table 4:** Reporting sources and patient outcomes for adverse events associated with denosumab and romosozumab.

Category	Specification	Denosumab, *n* (%)	Romosozumab, *n* (%)
Reporting source	Physician	11,256 (29.0)	4,244 (42.4)
	Consumer	14,117 (36.4)	2,730 (27.3)
	Health professional	7,910 (20.4)	1,863 (18.6)
	Pharmacist	3,146 (8.1)	1,002 (10.0)
	Missing / not specified	2,355 (6.1)	168 (1.7)
Patient outcome	Hospitalization	3,662 (9.4)	1,912 (19.1)
	Death	2,439 (6.3)	420 (4.2)
	Disability	538 (1.4)	79 (0.8)
	Life-threatening	127 (0.3)	56 (0.6)
	Congenital anomaly	3 (0.0)	1 (0.0)
	Other serious / required intervention	27,015 (69.7)	5,539 (55.4)

The outcome category “Other Serious / Required Intervention” includes events considered serious that did not result in hospitalization, death, disability, life-threatening illness, or congenital anomaly, but which required significant medical or surgical intervention to prevent such outcomes, in accordance with FAERS criteria. The reporting source category “Missing / Not Specified” includes reports where the reporter type was not provided or was uninterpretable.

### 3.4 Indications, time-to-onset, and frequently reported events

**Figure 3:**
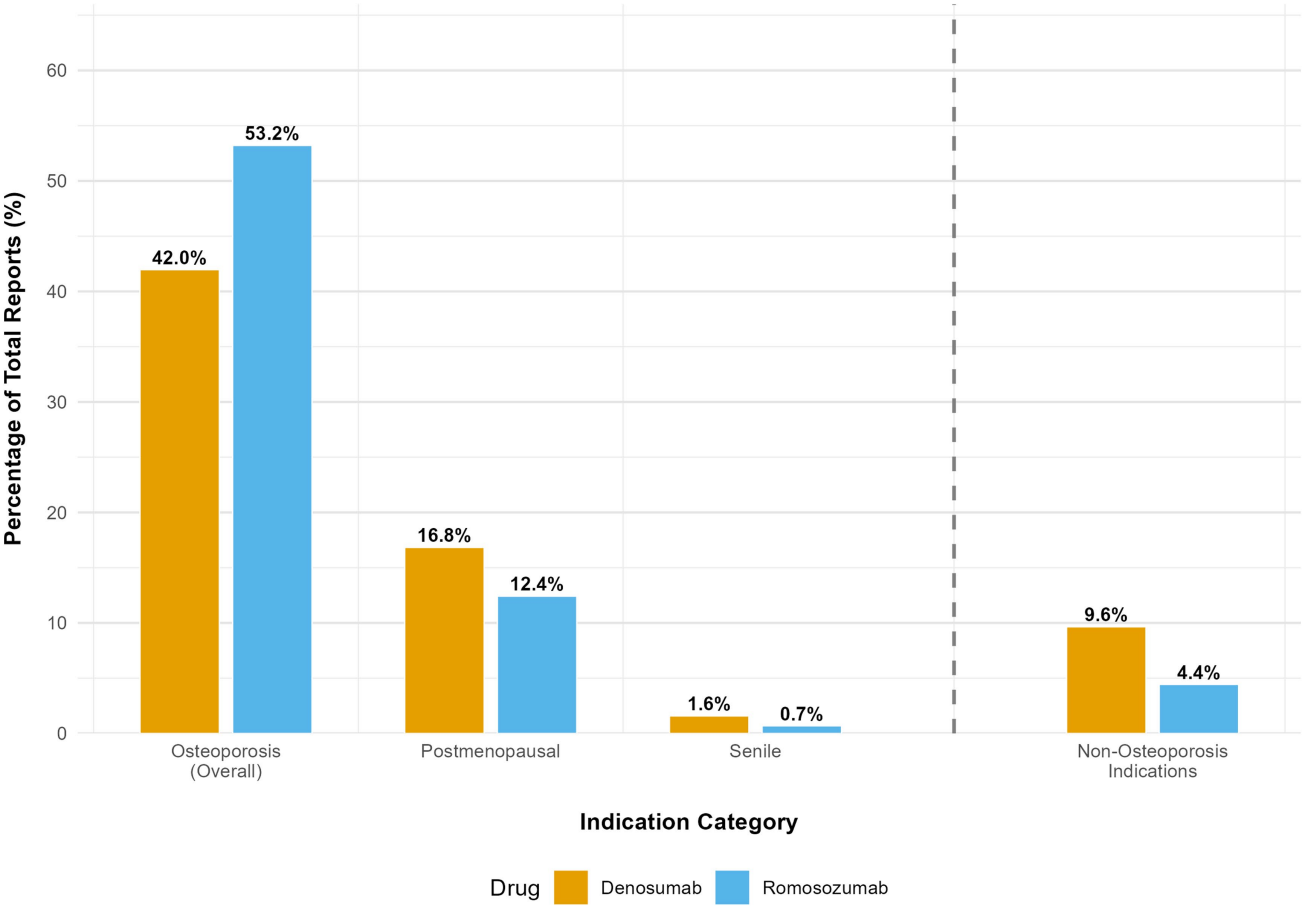
Primary reported indications for denosumab and romosozumab. The stacked bar chart demonstrates that the majority of adverse event reports for both drugs originated from their use in osteoporosis and its subtypes.

The primary indications for both drugs are summarized in Supplementary Table S1 and are visually compared in Figure 3. Most reports with specified indications involved the treatment of osteoporosis and its subtypes (e.g., postmenopausal and senile). When combined, osteoporosis-related indications accounted for 42.0% of the reports for denosumab and 53.2% for romosozumab, confirming that the analyzed safety data primarily reflected the intended therapeutic use. Among the non-osteoporosis indications, denosumab demonstrated considerable use in oncology settings (e.g., bone metastases and breast cancer), consistent with its approved label, whereas non-osteoporosis indications for romosozumab were infrequent and likely reflected off-label use, reporting errors, or patient comorbidities.

Most adverse events related to denosumab treatment occurred more than 360 days after administration (44.9%), whereas only 25.3% occurred within 30 days. This suggests that the adverse effects may be related to cumulative exposure and prolonged inhibition of bone turnover. In contrast, adverse events associated with romosozumab were concentrated within the first 30 days (36.0%) and only 3.1% occurred after 360 days (Table 5). These temporal clusters may be related to the acute reactions associated with rapid anabolic activity.

**Table 5:** Time-to-onset analysis of adverse events for denosumab and romosozumab.

Time-to-onset (days)	Denosumab		Romosozumab	
	*n*	%	*n*	%
0–30	844	25.29	479	36.02
31–60	178	5.33	186	13.98
61–90	178	5.33	142	10.68
91–120	85	2.55	98	7.37
121–150	71	2.13	70	5.26
151–180	115	3.45	65	4.89
181–360	427	12.80	249	18.72
>360	1,498	44.89	41	3.08
Total (with TTO data)	3,396	100.0	1,330	100.0

To elucidate the clinical implications of the distinct temporal patterns, we analyzed adverse events stratified by time-to-onset (≤30 days vs. >360 days). The drugs exhibited contrasting risk profiles (Supplementary Table S2). The early events of romosozumab were highly concentrated in acute cardiovascular and cerebrovascular HLT(High-Level Term)s, including *central nervous system hemorrhage*, *cerebrovascular accidents*, *ischemic coronary artery disorders*, and *heart failure*. *Injection site reactions* were also notable, underscoring the acute risk pattern. In contrast, late events associated with denosumab treatment were characterized by cumulative skeletal and metabolic HLTs. The most prominent disorders were *bone disorders neck*, *limb fractures and dislocations*, *spinal fractures and dislocations*, and *calcium metabolism disorders*. *Dental and oral soft-tissue infections* were also observed. This profile aligns with the mechanism of the prolonged suppression of bone turnover. This time-stratified analysis clarified the specific nature of early and late risk by providing differential monitoring strategies.

Supplementary Table S3 lists the 10 most frequently reported adverse events at the Preferred Term level. The most frequently reported adverse events associated with denosumab were product storage errors (*n* = 3,179) and death (*n* = 2,382), whereas the most frequently reported adverse events associated with romosozumab were fractures (*n* = 565) and falls (*n* = 565). Denosumab may be stored at temperatures ≤ 25 °C for as long as 7 d during a single usage cycle, a practice intended to improve convenience; however, it may also lead to misunderstanding and negligence. The reasons for the high ranking of serious adverse events (deaths, fractures, and falls) must be interpreted with caution, as most patients were older adults with osteoporosis and multiple comorbidities (e.g., cardiovascular or cerebrovascular disease and renal insufficiency). Poor underlying health is likely to increase the risk of fractures, falls, and death over a long course of treatment. These outcomes may be due to progression of the underlying disease itself rather than being directly caused by the drug, although such conditions may be reported together.

### 3.5 Signal detection and refinement at the high-level term

Both frequentist (ROR and PRR) and Bayesian (EBGM and BCPNN) frameworks were used to screen all Preferred Term level adverse event signals (lower limit of 95% confidence interval > 1; a ≥ 3; PRR ≥ 2; *χ*^2^ ≥ 4; EBGM₀₅ > 2; IC₀₂₅ > 0). In total, 410 and 197 potential signals corresponded to denosumab and romosozumab, respectively. Due to the large number of signals, we aggregated the events to the HLT level and identified 24 HLT signals for denosumab and 22 for romosozumab (Table 6).

**Table 6:** Validated safety signals at the High-Level Term (HLT) for denosumab and romosozumab.

System organ class / high-level term (HLT)	Denosumab		Romosozumab	
	EBGM (EBGM₀₅)	IC (IC₀₂₅)	EBGM (EBGM₀₅)	IC (IC₀₂₅)
Musculoskeletal, connective tissue, and metabolic disorders				
Fractures	46.54	5.42	35.67	4.66
Bone and joint injuries	13.41	3.74	14.42	3.84
Bone disorders (excluding congenital and fractures)	11.55	3.53	3.87	1.94
Bone, calcium, magnesium, and phosphorus metabolism disorders	14.12	3.80	4.22	2.01
Skeletal neoplasms malignant and unspecified	18.77	4.14	—	—
Skeletal neoplasms benign	13.93	2.98	—	—
Musculoskeletal and connective tissue disorders neck	2.95	1.56	2.02	1.01
Muscle disorders	2.03	1.02	—	—
Musculoskeletal and connective tissue deformities (including intervertebral disc disorders)	2.46	1.29	—	—
Bone and joint therapeutic procedures	3.01	1.58	—	—
Musculoskeletal and soft tissue investigations (excluding enzyme tests)	5.55	2.46	20.69	4.31
Water, electrolyte, and mineral investigations	3.62	1.85	2.23	1.13
Therapeutic procedures				
Head and neck therapeutic procedures	28.00	4.78	5.87	2.45
Nervous system, skull, and spine therapeutic procedures	2.73	1.42	3.39	1.66
Therapeutic procedures and supportive care neck	—	—	2.44	1.28
Endocrine gland therapeutic procedures	5.54	2.26	3.49	1.10
Cardiovascular and cerebrovascular disorders				
Coronary artery disorders	—	—	5.14	2.34
Heart failures	—	—	4.17	2.03
Aneurysms and artery dissections	—	—	8.06	2.75
Central nervous system vascular disorders	—	—	4.48	2.15
Cardiac valve disorders	—	—	2.98	1.48
Endocrine, neoplasms, and investigations				
Parathyroid gland disorders	7.24	2.75	—	—
Endocrine neoplasms benign	4.65	2.00	—	—
Neoplastic and ectopic endocrinopathies	5.33	1.80	—	—
Gastrointestinal neoplasms benign	2.15	0.84	—	—
Metastases	4.30	2.09	—	—
Endocrine investigations (including sex hormones)	2.90	1.52	—	—
Lipid analyses	2.10	1.06	—	—
Nervous system disorders				
Spinal cord and nerve root disorders	3.34	1.71	—	—
Other disorders				
Dental and gingival conditions	11.55	3.52	5.35	2.39
Tissue disorders neck	2.34	1.22	—	—
Administration site reactions	—	—	4.04	2.01
Injuries neck	—	—	2.57	1.35
Enzyme investigations neck	—	—	3.13	1.59

The EBGM and IC values are presented here as representative measures of signal strength and statistical confidence.

**Figure 4:**
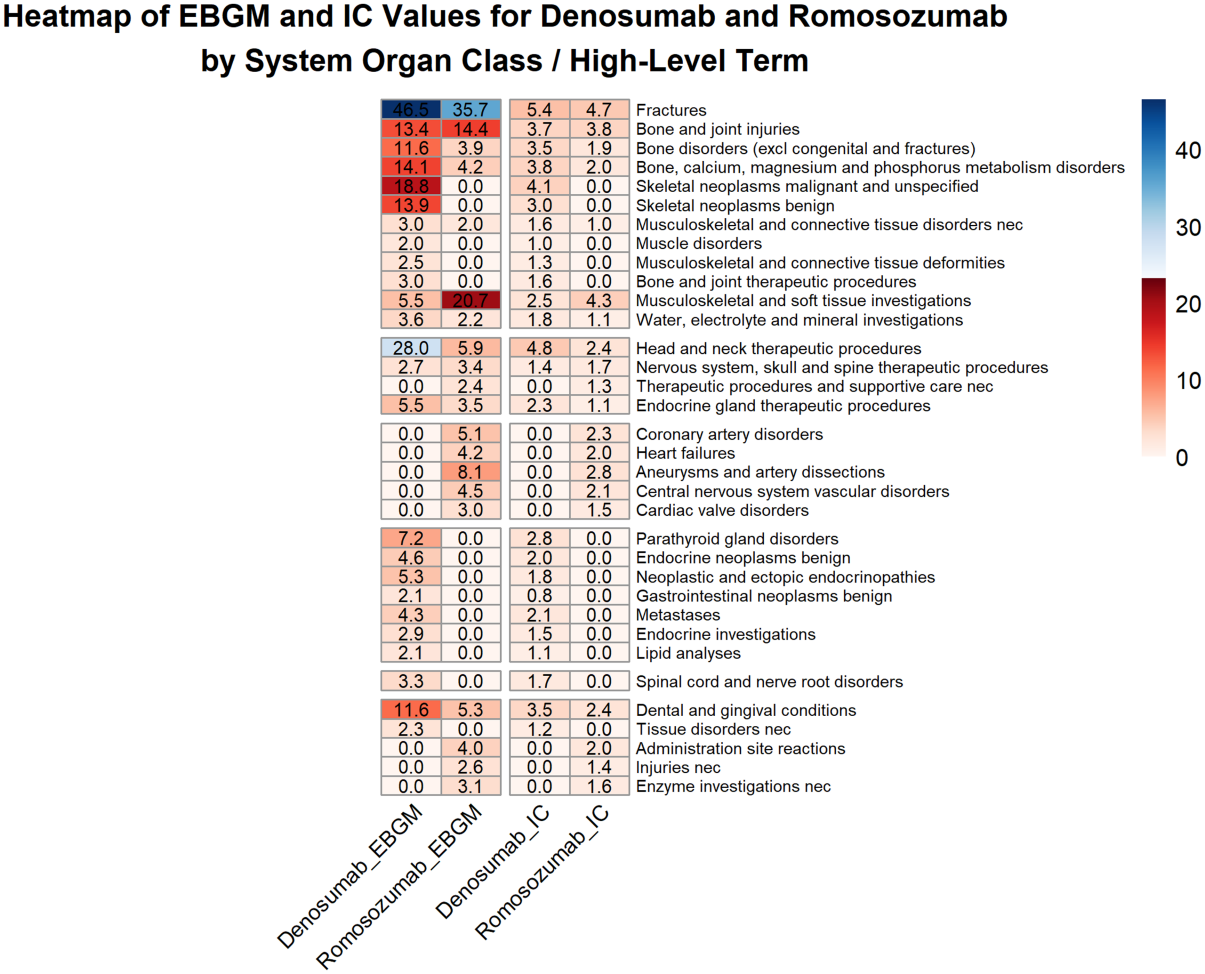
Heatmap analysis of disproportionality signals for denosumab and romosozumab across system organ classes/high-level terms.

Based on these HLT-level signals, denosumab exhibits various adverse effects. Strong signals included *bone, calcium, magnesium*, *and phosphorus metabolism disorders* (EBGM₀₅ = 14.12) and *bone, joint injuries* (EBGM₀₅ = 13.41), as well as *skeletal neoplasms malignant and unspecified* (EBGM₀₅ = 18.77) and s*keletal neoplasms benign* (EBGM₀₅ = 13.93). However, as shown in Supplementary Table S1, denosumab has been approved for use in patients with cancer (e.g., bone metastases); therefore, these signals should be interpreted cautiously. In contrast, romosozumab treatment resulted in a more concentrated HLT profile. *Coronary artery disorders* (EBGM₀₅ = 5.14), h*eart failure* (EBGM₀₅ = 4.17), and *aneurysms and artery dissections* (EBGM₀₅ = 8.06) were the principal cardiovascular concerns, providing data-driven support for early warning of cardiovascular risk. The signal for *central nervous system vascular disorders* (EBGM₀₅ = 4.48) logically extends cardiovascular risk to the cerebrovascular system. The relative strengths of the signals are shown in Figure 4.

### 3.6 Insights from system organ class analysis

The HLT signals were mapped to their corresponding system organ classes (SOCs) to provide a system-level perspective (Supplementary Table S4). The SOC profile associated with denosumab reflected long-term, cumulative systemic effects, with strong signals observed in *musculoskeletal and connective tissue disorders* (EBGM₀₅ = 3.26), *surgical and medical procedures* (EBGM₀₅ = 2.42), and *metabolism and nutrition disorders* (EBGM₀₅ = 1.22).

**Figure 5:**
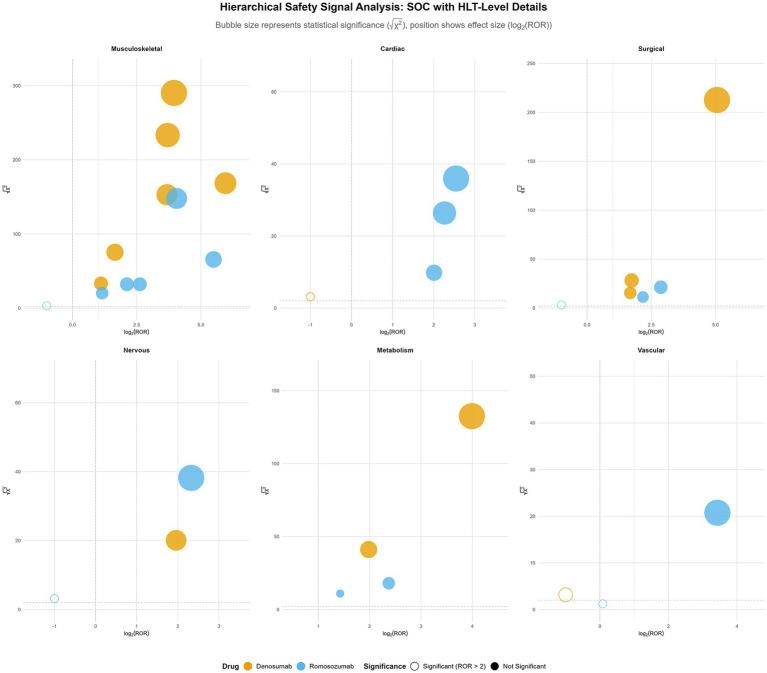
Hierarchical safety signal analysis across system organ classes. Bubble plots display high-level terms (HLTs) within six representative system organ classes (SOCs), with bubble position indicating effect size (log₂-transformed reporting odds ratio) and statistical significance (square root of *χ*^2^ value). Bubble size corresponds to statistical significance √(*χ*^2^), color denotes drug (orange: denosumab, blue: romosozumab), and fill style indicates signal significance (solid: ROR > 2, hollow: ROR ≤ 2).

In contrast, the SOC profile of romosozumab reinforces its characterization as an agent with predominantly acute effects on the cardiovascular system. The strongest signal was observed in *cardiac disorders* (EBGM₀₅ = 2.61). Additionally, the signal in *nervous system disorders* (EBGM₀₅ = 1.13) consisted primarily of cerebrovascular events (*central nervous system vascular disorders*: log₂ ROR = 2.33; √(*χ*^2^) = 38.2). Figure 5 shows the hierarchical composition of each SOC.

An exploratory subgroup analysis suggested that the reporting proportion of cardiac disorders associated with romosozumab was numerically higher in older patients (particularly in those aged > 85 years) and male patients (Supplementary Table S5). This pattern, consistent with the baseline cardiovascular risk, highlights subgroups that may warrant intensified monitoring.

### 3.7 Analysis of immune-related adverse events

Using the FDA guidance document (17), we developed criteria to classify immune-related events. An HLT was classified as immune-related only if it contained one or more positive Preferred Term signals clearly identified in the FDA guidelines or medical consensus as immune-mediated (e.g., *noninfectious gingivitis* and *injection-site urticaria*). Based on these criteria, all the HLTs listed in Table 6 were reviewed, and all positive Preferred Term signals within each HLT were examined.

For denosumab, a clear immune-related signal was detected in the *dental and gingival conditions* HLT, supported by the Preferred Term *noninfectious gingivitis*, which directly indicates a local immune-inflammatory response. Additionally, the presence of the Preferred Term *steroid therapy* in *endocrine gland therapeutic procedures* HLT represents a highly suspicious indirect signal, suggesting inflammatory adverse events requiring immunosuppressive management in clinical practice. For romosozumab, the immune-related signal was concentrated within the *administration-site reactions* HLT, which includes several typical type I hypersensitivity-preferred terms, such as *injection-site urticaria* and *injection-site erythema*. Among the other reviewed HLTs, including those associated with cardiovascular and cerebrovascular events, the Preferred Terms did not clearly map to systemic immune-mediated diseases (Table 7).

**Table 7:** Screening of potential immune-related adverse events at the high-level term (HLT) for denosumab and romosozumab.

High-level term (HLT)	Classification	Key preferred term(s)	Rationale
Denosumab			
Dental and gingival conditions	Immune-related	Noninfective gingivitis	Local immune-mediated inflammation
Endocrine gland therapeutic procedures	Immune-related (Indirect)	Steroid therapy	Management of inflammatory event
Musculoskeletal and connective tissue disorders neck	Non-immune-related	Myalgia, musculoskeletal pain	Non-specific symptoms
Muscle disorders	Non-immune-related	Myalgia	Non-specific symptom
Romosozumab			
Administration site reactions	Immune-related	Injection site urticaria, injection site erythema	Type I hypersensitivity
Musculoskeletal and connective tissue disorders neck	Non-immune-related	Pain in extremity	Non-specific symptom
Coronary artery disorders	Non-immune-related	Myocardial infarction	Ischemic event
Central nervous system vascular disorders	Non-immune-related	Cerebrovascular accident	Ischemic event

## 4 Discussion

In recent years, denosumab and romosozumab have been widely used to treat osteoporosis and their safety has become a focus of research attention. Several randomized controlled trials have established the long-term anti-fracture efficacy of denosumab and reported potential risks of adverse events, such as hypocalcemia and osteonecrosis of the jaw (5, 18). Although the beneficial effects of romosozumab in promoting bone formation have been confirmed in multiple studies, the primary risk is cardiovascular safety (9, 19). Therefore, researchers have begun to compare the two drugs. Davis et al. published a comparative review (20) that systematically evaluated the efficacy and safety of these agents. However, existing comparative studies have mostly summarized previously published literature and lack head-to-head, comprehensive, and systematic comparisons of the safety profiles of these two biological agents.

Based on the FAERS database, this study presents the first systematic and direct comparison of the real-world safety profiles of denosumab and romosozumab. By integrating multiple signal detection algorithms, we verified the classical risks associated with both drugs and, more importantly, revealed their distinct risk patterns in terms of time dynamics, SOC, and immune specificity, thus providing an important new perspective for pharmacovigilance in precision medicine in clinical practice.

### 4.1 Population and report characteristics

Our data showed that both denosumab and romosozumab were predominantly used in older women, correctly reflecting the core patient population with osteoporosis (1). Regarding geographic distribution, romosozumab was reported in a much higher proportion in Japan (38.6%) than denosumab (6.5%). This finding may be related to the earlier approval of romosozumab in Japan, regional healthcare policies, and the prevailing clinical practice preferences. This observation aligns with the pattern of romosozumab use in the Asia-Pacific region reported by Miyauchi et al. (21).

In addition, the profiles of reporting sources and patient outcomes suggested differences in risk patterns between the two drugs. Romosozumab is reported more frequently by physicians and is associated with serious outcomes, leading to hospitalization approximately twice as often as with denosumab. This is consistent with the acute cardiovascular risk signal associated with romosozumab, as serious events are more likely to be identified and reported by healthcare professionals, resulting in hospital admission. By contrast, the long-term cumulative risks associated with denosumab, such as abnormalities in bone metabolism, may be more readily perceived and spontaneously reported by patients in the community.

### 4.2 Overall safety Spectrum and temporal dynamics characteristics

Denosumab has approximately four times as many reports as romosozumab, which is consistent with its longer market presence and wider range of indications. The adverse events associated with denosumab primarily occurred after 360 days of treatment, demonstrating typical long-term cumulative risk characteristics. In contrast, adverse events associated with romosozumab are highly concentrated within the first 30 days, reflecting an early acute risk pattern (22, 23). The long-term risk pattern of denosumab is consistent with the results of multiple long-term extension studies (24), whereas the acute risk pattern of romosozumab provides real-world confirmation of the cardiovascular risk signals observed in pivotal clinical trials (19).

### 4.3 Distribution of risks across systemic organ categories

Based on the strong HLT signals, SOC-level mapping further translated these temporal patterns into system-specific risk distributions. Denosumab demonstrated a broad and systemic risk spectrum; *musculoskeletal and connective tissue disorders*, *metabolism and nutrition disorders*, and *surgical and medical procedures* (derived from treatment complications such as osteonecrosis of the jaw) all showed strong signals (25). This aligns with the mechanism by which denosumab, a RANKL inhibitor, may lead to excessive inhibition of bone turnover by continuous suppression of bone resorption, thereby increasing the risk of atypical fractures and mineral metabolism disorders (5, 18, 26). Risk management involves long-term monitoring and sequential treatment following drug withdrawal (3).

In contrast, the risk spectrum for romosozumab is highly focused on *cardiac disorders* and *nervous system disorders*, with *nervous system disorders* primarily consisting of cerebrovascular events. Strong signs, such as *coronary artery disorders*, *heart failure*, and *central nervous system vascular disorders*, provide real-world support for acute cardiovascular and cerebrovascular risks associated with romosozumab (9, 27). The underlying mechanism may be related to the complex role of the Wnt/*β*-catenin signaling pathway in vascular homeostasis, atherosclerosis, and vascular calcification (10). As noted in a review by Davis et al. (20), clinicians must rigorously assess the baseline cardio-cerebrovascular status of patients before initiating romosozumab treatment.

### 4.4 Immune-related adverse events

Denosumab and romosozumab exhibit immune-related mechanisms of action. We conducted a comparative analysis of immune-related adverse events and no widespread or systemic immune-mediated disease signals were detected in either group. Mechanism-specific local immune signals were identified by precise HLT/PT mapping.

For denosumab, a clear immune-related signal was reflected in the d*ental and gingival conditions* of HLT, with *noninfectious gingivitis* serving as the primary evidence. This finding is not incidental, as RANKL is also expressed in immune cells such as T lymphocytes within gingival tissue, and denosumab may locally disrupt homeostasis within the oral immune microenvironment by inhibiting RANKL, thereby inducing inflammation (6, 7). In addition, the presence of *steroid therapy* within the e*ndocrine gland therapeutic procedures* of HLT represents a highly suspicious indirect signal, suggesting that some patients may experience adverse inflammatory events requiring glucocorticoid treatment in clinical practice. However, only three such reports were identified and no concomitant events were recorded. Consequently, although *steroid therapy* remains a noteworthy signal, its specific link to drug-induced immune events could not be further elucidated through co-occurrence analysis in this dataset.

For romosozumab, immune-related signals were clearly identified within the *administration site reactions* of HLT, which included typical type I hypersensitivity reactions, such as urticaria and erythema. These reactions are more likely to be immediate local allergic responses triggered by the protein structure of the drug rather than systemic immune effects related to sclerostin inhibition. This difference may reflect the distinct immunological roles of RANKL and sclerostin and may account for the different clinical manifestations and management strategies.

### 4.5 Limitations

This study has several limitations. First, spontaneous reporting systems inherently suffer from reporting bias, missing information, and an inability to calculate precise incidence rates (28, 29). Second, signal detection only indicates a statistical association; establishing causality requires epidemiological study designs and experimental validation. Third, underlying disease status serves as an important confounding factor. For example, the strong signal for *skeletal neoplasms malignant and unspecified* reported for denosumab was most likely due to its approval for use in patients with bone metastases from solid tumors rather than the tumorigenic properties of the drug itself (30). Future studies should validate the risk–benefit ratio in specific populations, such as patients of advanced age or those with cardiovascular disease, using data from electronic health records or prospective registries (31). Exploring the associations between biomarker levels such as sclerostin and RANKL and adverse events may help explain the safety signals observed in this study (32).

## 5 Conclusion

This analysis revealed distinct safety profiles. Denosumab is primarily characterized by skeletal and metabolic risks associated with long-term cumulative use, whereas romosozumab is characterized by early risks associated with acute cardiovascular and cerebrovascular events. Regarding immune-related adverse events, denosumab was associated with osteoimmune complications such as noninfectious gingivitis, whereas romosozumab was associated only with mild injection-site reactions.

These findings provide critical decision-making support for the individualized treatment of osteoporosis. Clinicians should perform comprehensive risk–benefit assessments when planning therapy. In patients with cardiovascular disease or an elevated cardiovascular risk, romosozumab should be used cautiously and cardiovascular and cerebrovascular monitoring should be strengthened at the initiation of treatment. For patients requiring long-term denosumab therapy, structured monitoring of bone and mineral metabolism and careful management after treatment discontinuation are essential.

## Data Availability

The original contributions presented in the study are included in the article/Supplementary material, further inquiries can be directed to the corresponding author.
