# Supplementary material for: Comparative safety of denosumab and romosozumab in osteoporosis: an analysis based on the FDA adverse event reporting system database
Source: Front Med (Lausanne). 2026 Feb 5;13:1766601. doi: 10.3389/fmed.2026.1766601 (PMC12916682; doi:10.3389/fmed.2026.1766601)
Supplement: Supplementary file 1 [file Table_1.DOCX]

Supplementary Table S1. Top 10 reported indications for denosumab and romosozumab.

| Rank | Denosumab | n | Romosozumab | n |
| --- | --- | --- | --- | --- |
| 1 | Drug use for unknown indication | 16,786 | Drug use for unknown indication | 4,670 |
| 2 | Osteoporosis | 9,136 | Osteoporosis | 4,013 |
| 3 | Not reported (Missing) | 8,676 | Not reported (Missing) | 1,629 |
| 4 | Postmenopausal osteoporosis | 6,529 | Postmenopausal osteoporosis | 1,243 |
| 5 | Bone metastases | 1,446 | Hypertension | 168 |
| 6 | Prophylaxis | 713 | Off label use | 110 |
| 7 | Senile osteoporosis | 614 | Osteoporotic fracture | 69 |
| 8 | Breast cancer | 599 | Rheumatoid arthritis | 66 |
| 9 | Osteopenia | 552 | Pain | 57 |
| 10 | Metastatic breast cancer | 432 | Constipation | 43 |
