# Supplementary material for: Comparative safety of denosumab and romosozumab in osteoporosis: an analysis based on the FDA adverse event reporting system database
Source: Front Med (Lausanne). 2026 Feb 5;13:1766601. doi: 10.3389/fmed.2026.1766601 (PMC12916682; doi:10.3389/fmed.2026.1766601)
Supplement: Supplementary file 2 [file Table_2.DOCX]

Supplementary Table S2. High-Level Term (HLT) Analysis of Adverse Events by Time-to-Onset

*HLTs with ≥3 reports in the specified time window are shown; % is calculated against total reports for that drug in that window.*

| Drug (Time Window) | Key HLT Categories (n, %) | Representative PTs | Risk Pattern Interpretation |
| --- | --- | --- | --- |
| **Romosozumab (≤30 days)** | **1. Cardiovascular/Cerebrovascular** • CNS haemorrhages & cerebrovascular accidents (28, 2.77%) • Ischaemic coronary artery disorders (24, 2.37%) • Heart failures nec (16, 1.58%) | Stroke, Myocardial infarction, Heart failure | **Acute vascular risk**, supporting need for early cardiovascular monitoring. |
|  | **2. Injection-site/Acute Reactions** • Injection site reactions (24, 2.37%) • Headaches nec (19, 1.88%) | Injection site pain, Headache | **Acute drug reactions**. |
| **Denosumab (>360 days)** | **1. Skeletal & Metabolic** • Bone disorders nec (273, 8.12%) • Limb fractures & dislocations (155, 4.61%) • Spinal fractures & dislocations (123, 3.66%) • Calcium metabolism disorders (39, 1.16%) | Hypocalcaemia, Atypical femoral fracture | **Long-term cumulative risk** from sustained bone turnover suppression. |
|  | **2. Jaw & Oral Events** • Dental & oral soft tissue infections (42, 1.25%) | Osteonecrosis of jaw | **Site-specific long-term risk** linked to immunomodulation in the oral microenvironment. |
