# Supplementary material for: Comparative safety of denosumab and romosozumab in osteoporosis: an analysis based on the FDA adverse event reporting system database
Source: Front Med (Lausanne). 2026 Feb 5;13:1766601. doi: 10.3389/fmed.2026.1766601 (PMC12916682; doi:10.3389/fmed.2026.1766601)
Supplement: Supplementary file 3 [file Table_3.DOCX]

Supplementary Table S3. Top 10 reported adverse events (Preferred Terms) for denosumab and romosozumab.

| Rank | Denosumab | n | Romosozumab | n |
| --- | --- | --- | --- | --- |
| 1 | Product storage error | 3,179 | Fall | 565 |
| 2 | Death | 2,382 | Fracture | 565 |
| 3 | Arthralgia | 1,958 | Injection site pain | 444 |
| 4 | Circumstance capable of leading to medication error | 1,918 | Bone density abnormal | 436 |
| 5 | Osteonecrosis of jaw | 1,777 | Arthralgia | 429 |
| 6 | Off label use | 1,624 | Off label use | 364 |
| 7 | Fall | 1,491 | Headache | 306 |
| 8 | Spinal fracture | 1,491 | Hospitalisation | 305 |
| 9 | Pain in extremity | 1,335 | Death | 249 |
| 10 | Back pain | 1,267 | Product storage error | 249 |
