# Supplementary material for: Comparative safety of denosumab and romosozumab in osteoporosis: an analysis based on the FDA adverse event reporting system database
Source: Front Med (Lausanne). 2026 Feb 5;13:1766601. doi: 10.3389/fmed.2026.1766601 (PMC12916682; doi:10.3389/fmed.2026.1766601)
Supplement: Supplementary file 4 [file Table_4.DOCX]

Supplementary Table S4. System Organ Class (SOC) level safety profiles for denosumab and romosozumab.

| System Organ Class (SOC) | Denosumab |  | Romosozumab |  |
| --- | --- | --- | --- | --- |
|  | EBGM (EBGM₀₅) | IC (IC₀₂₅) | EBGM (EBGM₀₅) | IC (IC₀₂₅) |
| **Musculoskeletal and connective tissue disorders** | 3.32 (3.26) | 1.73 (1.71) | 2.20 (2.10) | 1.14 (1.07) |
| **Injury, poisoning and procedural complications** | 1.90 (1.87) | 0.92 (0.90) | 1.60 (1.55) | 0.68 (0.63) |
| **Surgical and medical procedures** | 2.50 (2.42) | 1.32 (1.27) | 2.45 (2.28) | 1.29 (1.18) |
| **Metabolism and nutrition disorders** | 1.27 (1.22) | 0.34 (0.28) | 0.85 (0.77) | -0.23  (-0.39) |
| **Cardiac disorders** | 0.58 (0.54) | -0.79  (-0.88) | 2.78 (2.61) | 1.47 (1.38) |
| **Vascular disorders** | 0.56 (0.52) | -0.85  (-0.94) | 1.06 (0.96) | 0.09  (-0.06) |
| **Nervous system disorders** | 0.61 (0.59) | -0.71  (-0.76) | 1.19 (1.13) | 0.25 (0.18) |
| **General disorders and administration site conditions** | 0.72 (0.71) | -0.47  (-0.50) | 1.07 (1.03) | 0.10 (0.05) |
| **Investigations** | 0.89 (0.87) | -0.17  (-0.21) | 1.18 (1.12) | 0.24 (0.16) |
| **Neoplasms benign, malignant and unspecified** | 0.93 (0.90) | -0.10  (-0.16) | 0.44 (0.39) | -1.18  (-1.34) |

Note: Only SOCs with significant signals (EBGM₀₅ > 2 or notable patterns) are displayed. EBGM, Empirical Bayes Geometric Mean; IC, Information Component. The lower 95% confidence intervals (EBGM₀₅ and IC₀₂₅) are provided in parentheses. SOCs are ordered by the magnitude of denosumab signals.
