# Supplementary material for: Comparative safety of denosumab and romosozumab in osteoporosis: an analysis based on the FDA adverse event reporting system database
Source: Front Med (Lausanne). 2026 Feb 5;13:1766601. doi: 10.3389/fmed.2026.1766601 (PMC12916682; doi:10.3389/fmed.2026.1766601)
Supplement: Supplementary file 5 [file Table_5.DOCX]

Supplementary Table S5. Proportion of Cardiac Disorders Associated with Romosozumab, by Age and Sex

| Subgroup | Total Reports for Romosozumab (n) | Reports of Cardiac Disorders (SOC) (n) | Proportion (%) |
| --- | --- | --- | --- |
| **Age Group** |  |  |  |
| <65 years | 636 | 85 | 13.4% |
| 65-85 years | 2790 | 442 | 15.8% |
| >85 years | 673 | 161 | 23.9% |
| **Sex** |  |  |  |
| Female | 7036 | 823 | 11.7% |
| Male | 544 | 94 | 17.3% |
|  |  |  |  |
