# Supplementary figures and images for: Comparative safety of denosumab and romosozumab in osteoporosis: an analysis based on the FDA adverse event reporting system database
Source: Front Med (Lausanne). 2026 Feb 5;13:1766601. doi: 10.3389/fmed.2026.1766601 (PMC12916682; doi:10.3389/fmed.2026.1766601)

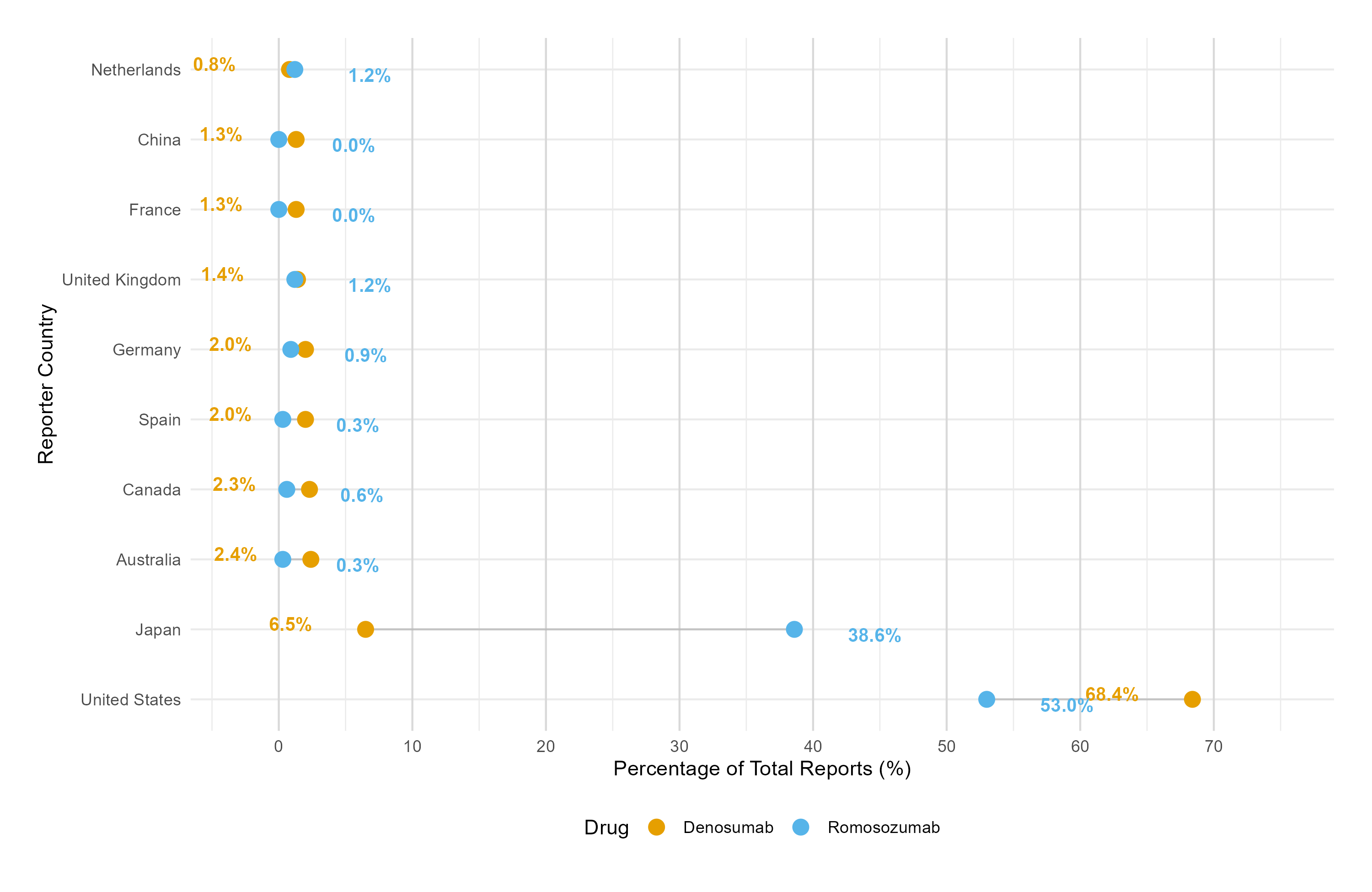

Supplement: Supplementary file 6 [file Image_1.TIFF]

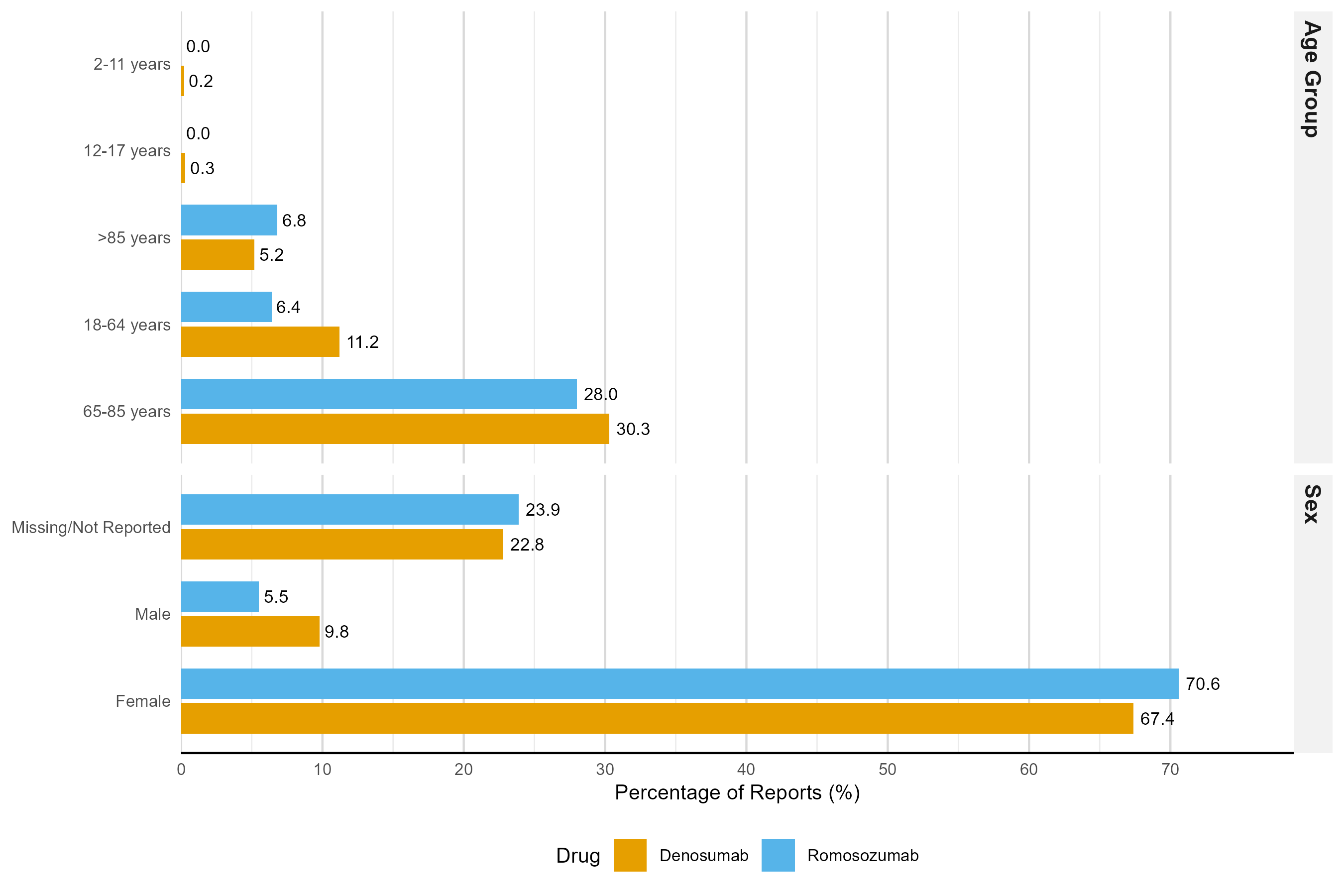

Supplement: Supplementary file 7 [file Image_2.TIFF]
